# *N*′-(4-Diethyl­amino-2-hy­droxy­benzyl­idene)-4-methyl­benzohydrazide

**DOI:** 10.1107/S1600536812010690

**Published:** 2012-03-17

**Authors:** Xi-Hai Shen, Li-Juen Shao, Zhao-Fu Zhu, Li-Xue Zhu

**Affiliations:** a Department of Chemistry, Hebei Normal University of Science and Technology, Qinhuangdao 066600, People’s Republic of China

## Abstract

The title compound, C_19_H_23_N_3_O_2_, was prepared by condensing 4-diethyl­amino-2-hy­droxy­benzaldehyde and 4-methyl­benzo­hydrazide in methanol. The asymmetric unit contains two independent mol­ecules in which the two benzene rings make dihedral angles of 30.3 (3) and 18.9 (3)°. Intra­molecular O—H⋯N hydrogen bonds are observed in both mol­ecules. The crystal structure is stabilized by N—H⋯O hydrogen bonds, which form chains along the *a* axis.

## Related literature

For the structures of similar hydrazone compounds, see: Fun *et al.* (2011 ▶); Horkaew *et al.* (2011 ▶); Zhi *et al.* (2011 ▶); Huang & Wu (2010 ▶); Shen *et al.* (2012 ▶). For standard bond lengths, see: Allen *et al.* (1987 ▶).
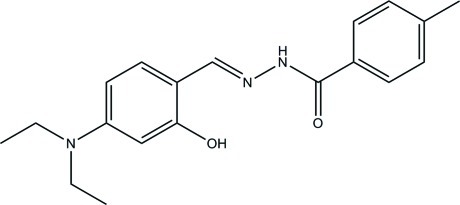


## Experimental

### 

#### Crystal data

C_19_H_23_N_3_O_2_
*M* *_r_* = 325.40
Triclinic, 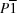

*a* = 9.923 (2) Å
*b* = 11.963 (2) Å
*c* = 15.827 (2) Å
α = 95.269 (2)°
β = 98.932 (2)°
γ = 103.691 (2)°
*V* = 1787.0 (5) Å^3^
*Z* = 4
Mo *K*α radiation
μ = 0.08 mm^−1^
*T* = 298 K
0.13 × 0.10 × 0.08 mm

#### Data collection

Bruker SMART CCD area-detector diffractometer
Absorption correction: multi-scan (*SADABS*; Bruker, 2001 ▶) *T*_min_ = 0.990, *T*_max_ = 0.994
13230 measured reflections
6512 independent reflections
1651 reflections with *I* > 2σ(*I*)
*R*_int_ = 0.137

#### Refinement

*R*[*F* ^2^ > 2σ(*F* ^2^)] = 0.086
*wR*(*F* ^2^) = 0.245
*S* = 0.85
6512 reflections
449 parameters
7 restraints
H atoms treated by a mixture of independent and constrained refinement
Δρ_max_ = 0.35 e Å^−3^
Δρ_min_ = −0.30 e Å^−3^

### 

Data collection: *SMART* (Bruker, 2007 ▶); cell refinement: *SAINT* (Bruker, 2007 ▶); data reduction: *SAINT*; program(s) used to solve structure: *SHELXTL* (Sheldrick, 2008 ▶); program(s) used to refine structure: *SHELXTL*; molecular graphics: *SHELXTL*; software used to prepare material for publication: *SHELXTL*.

## Supplementary Material

Crystal structure: contains datablock(s) global, I. DOI: 10.1107/S1600536812010690/sj5209sup1.cif

Structure factors: contains datablock(s) I. DOI: 10.1107/S1600536812010690/sj5209Isup2.hkl

Supplementary material file. DOI: 10.1107/S1600536812010690/sj5209Isup3.cml

Additional supplementary materials:  crystallographic information; 3D view; checkCIF report

## Figures and Tables

**Table 1:** Hydrogen-bond geometry (Å, °)

*D*—H⋯*A*	*D*—H	H⋯*A*	*D*⋯*A*	*D*—H⋯*A*
N2—H2⋯O4^i^	0.90 (1)	1.95 (2)	2.831 (7)	167 (6)
O1—H1⋯N1	0.82	1.93	2.641 (7)	145
N5—H5⋯O2	0.90 (1)	2.12 (2)	2.985 (7)	160 (6)
O3—H3⋯N4	0.85 (1)	1.94 (1)	2.581 (7)	132 (2)

Symmetry code: (i) 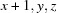.

**Fig. 1.**
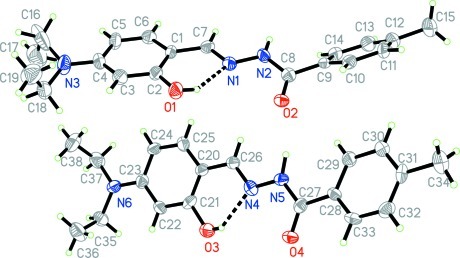
The molecular structure of (I) with ellipsoids drawn at the 30% probability level.

**Fig. 2.**
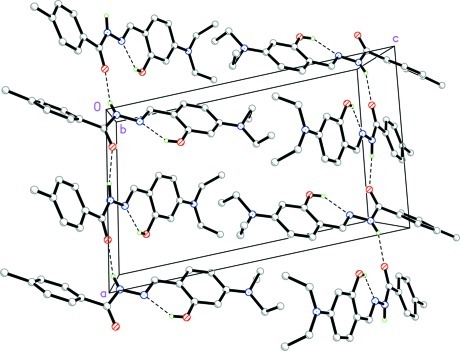
The crystal structure of (I). Hydrogen bonds are drawn as dashed lines.

## supplementary crystallographic information

### Comment

In the last few years, a number of hydrazone compounds have been reported (Fun *et al.*, 2011; Horkaew *et al.*, 2011; Zhi *et al.*, 2011; Huang & Wu, 2010). As an extension of our work on such compounds (Shen *et al.*, 2012), we report here the structure of a new benzohydrazide compound, (I).

The asymmetric unit of the compound contains two independent molecules (Fig.1) both of which form intramolecular O—H···N hydrogen bonds (Table 1). The dihedral angle between the C7-N1-N2-C8-O2 plane and the C1—C6 benzene ring is 15.4 (2)° while that between the C26-N4-N5-C27-O4 section of the molecule and the C20—C25 benzene ring is 5.8 (2)°. The planarity of these portions of the molecule may result from the formation of intramolecular O—H···N hydrogen bonds. All the bond distances are within normal ranges (Allen *et al.*, 1987) and comparable with those in the similar compounds reported recently and mentioned previously. The crystal structure of the compound is stabilized by intermolecular N—H···O hydrogen bonds, to form chains along the *a* axis (Table 1, Fig. 2).

### Experimental

2-Hydroxy-4-diethylaminobenzaldehyde (193.0 mg, 1.0 mmol) and 4-methylbenzohydrazide (150.1 mg, 1.0 mmol) were mixed in methanol (60 ml). The mixture was refluxed for 30 min, then cooled to room temperature, yielding a colorless solution. Small, colorless crystals were formed when the solution was evaporated in air for several days.

### Refinement

Hydrogen atoms bound to N and O were located in a difference Fourier map and refined isotropically, with N—H and O—H distances restrained to 0.90 (1) and 0.85 (1) Å. The remaining H atoms were placed in idealized positions and constrained to ride on their parent atoms, with C—H distances of 0.93–0.97 Å, and with *U*_iso_(H) set at 1.2*U*_eq_(C) and 1.5*U*_eq_(methyl C). High atomic displacement parameters for atom C16 indicated possible disorder. However a suitable model could not be developed and bond distances within the N3 C16 C17 unit were constrained using DFIX. Crystals were very small and weakly diffracting, which results in a very low ratio of observed/unique reflections.

### Crystal data

**Table:**

C_19_H_23_N_3_O_2_	*Z* = 4
*M**_r_* = 325.40	*F*(000) = 696
Triclinic, *P*1	*D*_x_ = 1.209 Mg m^−^^3^
Hall symbol: -P 1	Mo *K*α radiation, λ = 0.71073 Å
*a* = 9.923 (2) Å	Cell parameters from 358 reflections
*b* = 11.963 (2) Å	θ = 2.3–23.7°
*c* = 15.827 (2) Å	µ = 0.08 mm^−^^1^
α = 95.269 (2)°	*T* = 298 K
β = 98.932 (2)°	Block, colorless
γ = 103.691 (2)°	0.13 × 0.10 × 0.08 mm
*V* = 1787.0 (5) Å^3^	

### Data collection

**Table:**

Bruker SMART CCD area-detector diffractometer	6512 independent reflections
Radiation source: fine-focus sealed tube	1651 reflections with *I* > 2σ(*I*)
Graphite monochromator	*R*_int_ = 0.137
ω scans	θ_max_ = 25.5°, θ_min_ = 1.3°
Absorption correction: multi-scan (*SADABS*; Bruker, 2001)	*h* = −12→12
*T*_min_ = 0.990, *T*_max_ = 0.994	*k* = −14→14
13230 measured reflections	*l* = −19→19

### Refinement

**Table:**

Refinement on *F*^2^	Primary atom site location: structure-invariant direct methods
Least-squares matrix: full	Secondary atom site location: difference Fourier map
*R*[*F*^2^ > 2σ(*F*^2^)] = 0.086	Hydrogen site location: inferred from neighbouring sites
*wR*(*F*^2^) = 0.245	H atoms treated by a mixture of independent and constrained refinement
*S* = 0.85	*w* = 1/[σ^2^(*F*_o_^2^) + (0.0734*P*)^2^] where *P* = (*F*_o_^2^ + 2*F*_c_^2^)/3
6512 reflections	(Δ/σ)_max_ = 0.002
449 parameters	Δρ_max_ = 0.35 e Å^−^^3^
7 restraints	Δρ_min_ = −0.30 e Å^−^^3^

### Special details

**Table:**

Geometry. All esds (except the esd in the dihedral angle between two l.s. planes) are estimated using the full covariance matrix. The cell esds are taken into account individually in the estimation of esds in distances, angles and torsion angles; correlations between esds in cell parameters are only used when they are defined by crystal symmetry. An approximate (isotropic) treatment of cell esds is used for estimating esds involving l.s. planes.
Refinement. Refinement of F^2^ against ALL reflections. The weighted R-factor wR and goodness of fit S are based on F^2^, conventional R-factors R are based on F, with F set to zero for negative F^2^. The threshold expression of F^2^ > 2sigma(F^2^) is used only for calculating R-factors(gt) etc. and is not relevant to the choice of reflections for refinement. R-factors based on F^2^ are statistically about twice as large as those based on F, and R- factors based on ALL data will be even larger.

### Fractional atomic coordinates and isotropic or equivalent isotropic displacement parameters (Å^2^)

**Table:**

	*x*	*y*	*z*	*U*_iso_*/*U*_eq_	
N1	0.9155 (5)	0.6833 (5)	0.8899 (4)	0.0555 (16)	
N2	0.9803 (6)	0.7347 (5)	0.9734 (4)	0.0548 (16)	
N3	0.7708 (8)	0.4048 (6)	0.5086 (4)	0.091 (3)	
N4	0.4397 (6)	0.6246 (5)	0.9527 (3)	0.0497 (15)	
N5	0.4905 (5)	0.7327 (5)	1.0014 (4)	0.0465 (14)	
N6	0.3516 (6)	0.1601 (5)	0.6941 (4)	0.0610 (17)	
O1	0.7408 (5)	0.6651 (4)	0.7424 (3)	0.0652 (14)	
H1	0.7676	0.6823	0.7947	0.098*	
O2	0.7941 (5)	0.8102 (4)	0.9873 (3)	0.0597 (14)	
O3	0.2415 (5)	0.4445 (4)	0.8802 (3)	0.0631 (14)	
O4	0.2712 (5)	0.7437 (4)	1.0126 (3)	0.0649 (15)	
C1	0.9216 (7)	0.5602 (6)	0.7634 (4)	0.0473 (18)	
C2	0.8067 (8)	0.5856 (6)	0.7119 (4)	0.0526 (19)	
C3	0.7584 (7)	0.5351 (6)	0.6274 (5)	0.059 (2)	
H3A	0.6860	0.5570	0.5937	0.071*	
C4	0.8177 (8)	0.4515 (6)	0.5924 (5)	0.063 (2)	
C5	0.9315 (8)	0.4241 (6)	0.6437 (4)	0.066 (2)	
H5A	0.9735	0.3692	0.6213	0.080*	
C6	0.9797 (7)	0.4776 (6)	0.7254 (5)	0.059 (2)	
H6	1.0554	0.4584	0.7579	0.071*	
C7	0.9772 (7)	0.6152 (6)	0.8517 (4)	0.0527 (19)	
H7	1.0588	0.6011	0.8813	0.063*	
C8	0.9117 (8)	0.8019 (6)	1.0165 (4)	0.0504 (19)	
C9	0.9927 (7)	0.8600 (6)	1.1021 (4)	0.0480 (18)	
C10	0.9922 (7)	0.9737 (6)	1.1256 (5)	0.056 (2)	
H10	0.9404	1.0101	1.0881	0.068*	
C11	1.0673 (8)	1.0348 (6)	1.2039 (5)	0.065 (2)	
H11	1.0656	1.1116	1.2180	0.078*	
C12	1.1440 (8)	0.9839 (8)	1.2609 (5)	0.073 (2)	
C13	1.1380 (8)	0.8669 (7)	1.2397 (5)	0.069 (2)	
H13	1.1831	0.8284	1.2790	0.083*	
C14	1.0655 (7)	0.8080 (6)	1.1605 (5)	0.062 (2)	
H14	1.0660	0.7309	1.1465	0.075*	
C15	1.2289 (8)	1.0512 (6)	1.3466 (4)	0.094 (3)	
H15A	1.3126	1.1038	1.3366	0.141*	
H15B	1.2548	0.9979	1.3839	0.141*	
H15C	1.1728	1.0943	1.3732	0.141*	
C16	0.8588 (10)	0.3486 (8)	0.4632 (6)	0.151 (5)	
H16A	0.8486	0.3637	0.4037	0.182*	
H16B	0.9571	0.3788	0.4903	0.182*	
C17	0.8153 (10)	0.2270 (8)	0.4666 (6)	0.135 (4)	
H17A	0.8250	0.2130	0.5257	0.202*	
H17B	0.8732	0.1884	0.4376	0.202*	
H17C	0.7185	0.1977	0.4387	0.202*	
C18	0.6394 (8)	0.4169 (7)	0.4535 (5)	0.078 (2)	
H18A	0.5719	0.4274	0.4898	0.094*	
H18B	0.5976	0.3462	0.4136	0.094*	
C19	0.6687 (9)	0.5177 (7)	0.4035 (5)	0.110 (3)	
H19A	0.7166	0.5870	0.4425	0.166*	
H19B	0.5814	0.5272	0.3732	0.166*	
H19C	0.7271	0.5035	0.3628	0.166*	
C20	0.4824 (8)	0.4717 (6)	0.8644 (4)	0.0478 (18)	
C21	0.3419 (8)	0.4062 (6)	0.8446 (4)	0.0521 (19)	
C22	0.2982 (7)	0.3076 (6)	0.7870 (4)	0.0521 (19)	
H22	0.2025	0.2706	0.7721	0.063*	
C23	0.3943 (8)	0.2620 (6)	0.7503 (4)	0.056 (2)	
C24	0.5391 (7)	0.3225 (6)	0.7723 (4)	0.0548 (19)	
H24	0.6070	0.2936	0.7496	0.066*	
C25	0.5766 (7)	0.4243 (5)	0.8276 (4)	0.0525 (19)	
H25	0.6716	0.4638	0.8411	0.063*	
C26	0.5295 (7)	0.5816 (6)	0.9197 (4)	0.0465 (18)	
H26	0.6246	0.6210	0.9313	0.056*	
C27	0.3997 (8)	0.7875 (6)	1.0303 (4)	0.0487 (18)	
C28	0.4626 (7)	0.9045 (6)	1.0813 (4)	0.0440 (17)	
C29	0.6076 (8)	0.9461 (6)	1.1139 (4)	0.060 (2)	
H29	0.6682	0.8997	1.1042	0.072*	
C30	0.6610 (8)	1.0558 (6)	1.1605 (4)	0.063 (2)	
H30	0.7575	1.0828	1.1813	0.075*	
C31	0.5727 (9)	1.1254 (6)	1.1764 (4)	0.057 (2)	
C32	0.4292 (9)	1.0833 (6)	1.1442 (5)	0.063 (2)	
H32	0.3675	1.1287	1.1541	0.076*	
C33	0.3786 (7)	0.9747 (6)	1.0976 (4)	0.0529 (19)	
H33	0.2822	0.9482	1.0764	0.063*	
C34	0.6319 (8)	1.2448 (6)	1.2281 (4)	0.087 (3)	
H34A	0.6889	1.2944	1.1957	0.130*	
H34B	0.6885	1.2388	1.2816	0.130*	
H34C	0.5557	1.2770	1.2397	0.130*	
C35	0.2048 (8)	0.0962 (6)	0.6689 (5)	0.076 (2)	
H35A	0.1586	0.1014	0.7183	0.091*	
H35B	0.2002	0.0150	0.6528	0.091*	
C36	0.1260 (9)	0.1388 (8)	0.5955 (5)	0.118 (3)	
H36A	0.1224	0.2170	0.6127	0.177*	
H36B	0.0317	0.0898	0.5796	0.177*	
H36C	0.1731	0.1370	0.5470	0.177*	
C37	0.4532 (8)	0.1073 (6)	0.6589 (5)	0.080 (2)	
H37A	0.4124	0.0243	0.6448	0.096*	
H37B	0.5367	0.1193	0.7030	0.096*	
C38	0.4959 (9)	0.1541 (8)	0.5807 (5)	0.122 (4)	
H38A	0.4136	0.1455	0.5373	0.183*	
H38B	0.5576	0.1122	0.5590	0.183*	
H38C	0.5443	0.2348	0.5952	0.183*	
H2	1.068 (3)	0.726 (5)	0.988 (4)	0.080*	
H5	0.580 (2)	0.773 (5)	1.003 (4)	0.080*	
H3	0.2612 (18)	0.512 (2)	0.909 (4)	0.080*	

### Atomic displacement parameters (Å^2^)

**Table:**

	*U*^11^	*U*^22^	*U*^33^	*U*^12^	*U*^13^	*U*^23^
N1	0.044 (4)	0.066 (4)	0.054 (4)	0.018 (3)	0.005 (3)	−0.003 (3)
N2	0.036 (4)	0.067 (4)	0.056 (4)	0.016 (3)	−0.002 (3)	−0.012 (3)
N3	0.092 (6)	0.135 (7)	0.063 (5)	0.074 (5)	0.010 (5)	−0.019 (5)
N4	0.054 (4)	0.041 (4)	0.050 (4)	0.011 (3)	−0.002 (3)	0.007 (3)
N5	0.040 (4)	0.052 (4)	0.049 (4)	0.009 (3)	0.012 (3)	0.012 (3)
N6	0.053 (4)	0.066 (4)	0.063 (4)	0.013 (4)	0.019 (4)	−0.003 (3)
O1	0.065 (4)	0.071 (3)	0.061 (3)	0.032 (3)	0.005 (3)	−0.010 (3)
O2	0.036 (3)	0.076 (3)	0.064 (3)	0.016 (3)	0.005 (3)	−0.006 (3)
O3	0.051 (3)	0.061 (3)	0.072 (4)	0.011 (3)	0.010 (3)	−0.007 (3)
O4	0.035 (3)	0.077 (4)	0.077 (4)	0.013 (3)	0.005 (3)	−0.009 (3)
C1	0.045 (5)	0.060 (5)	0.038 (4)	0.016 (4)	0.008 (4)	−0.003 (4)
C2	0.058 (5)	0.053 (5)	0.046 (5)	0.008 (4)	0.017 (4)	0.003 (4)
C3	0.054 (5)	0.066 (5)	0.058 (5)	0.022 (4)	0.003 (4)	0.012 (4)
C4	0.070 (6)	0.070 (6)	0.040 (5)	0.015 (5)	0.001 (4)	−0.008 (4)
C5	0.070 (6)	0.091 (6)	0.041 (5)	0.032 (5)	0.003 (4)	−0.003 (4)
C6	0.044 (5)	0.080 (5)	0.059 (5)	0.031 (4)	0.005 (4)	0.008 (4)
C7	0.040 (5)	0.068 (5)	0.054 (5)	0.018 (4)	0.011 (4)	0.008 (4)
C8	0.051 (5)	0.046 (5)	0.053 (5)	0.008 (4)	0.009 (4)	0.011 (4)
C9	0.028 (4)	0.069 (5)	0.042 (4)	0.005 (4)	0.008 (4)	−0.004 (4)
C10	0.049 (5)	0.059 (5)	0.065 (5)	0.019 (4)	0.017 (4)	0.004 (4)
C11	0.067 (6)	0.054 (5)	0.063 (5)	−0.001 (4)	0.021 (5)	−0.016 (5)
C12	0.055 (6)	0.095 (7)	0.051 (5)	0.000 (5)	0.002 (4)	−0.009 (5)
C13	0.061 (6)	0.086 (6)	0.059 (6)	0.019 (5)	0.005 (5)	0.008 (5)
C14	0.048 (5)	0.076 (6)	0.063 (5)	0.018 (4)	0.012 (4)	0.003 (5)
C15	0.091 (7)	0.114 (7)	0.056 (5)	0.005 (6)	0.001 (5)	−0.012 (5)
C16	0.188 (13)	0.106 (9)	0.091 (8)	−0.036 (9)	−0.081 (8)	0.031 (7)
C17	0.149 (10)	0.135 (9)	0.109 (8)	0.034 (8)	−0.008 (7)	0.016 (7)
C18	0.069 (6)	0.100 (7)	0.063 (5)	0.033 (5)	0.000 (5)	−0.007 (5)
C19	0.111 (8)	0.144 (9)	0.095 (7)	0.048 (7)	0.035 (6)	0.045 (7)
C20	0.046 (5)	0.047 (5)	0.050 (5)	0.008 (4)	0.015 (4)	0.006 (4)
C21	0.060 (6)	0.067 (5)	0.046 (5)	0.034 (5)	0.024 (4)	0.019 (4)
C22	0.046 (5)	0.049 (5)	0.054 (5)	0.006 (4)	0.003 (4)	−0.003 (4)
C23	0.062 (6)	0.053 (5)	0.052 (5)	0.015 (5)	0.013 (4)	0.001 (4)
C24	0.046 (5)	0.054 (5)	0.065 (5)	0.013 (4)	0.019 (4)	−0.002 (4)
C25	0.058 (5)	0.050 (5)	0.043 (4)	0.004 (4)	0.006 (4)	0.005 (4)
C26	0.042 (5)	0.060 (5)	0.041 (4)	0.014 (4)	0.008 (4)	0.016 (4)
C27	0.057 (5)	0.058 (5)	0.037 (4)	0.020 (5)	0.012 (4)	0.013 (4)
C28	0.035 (5)	0.050 (5)	0.042 (4)	0.007 (4)	−0.001 (4)	0.007 (4)
C29	0.054 (6)	0.067 (5)	0.060 (5)	0.028 (4)	0.003 (4)	−0.008 (4)
C30	0.054 (5)	0.078 (6)	0.050 (5)	0.014 (5)	0.004 (4)	−0.002 (4)
C31	0.083 (6)	0.049 (5)	0.037 (4)	0.021 (5)	0.012 (5)	−0.005 (4)
C32	0.063 (6)	0.058 (5)	0.076 (6)	0.028 (5)	0.022 (5)	0.001 (4)
C33	0.029 (4)	0.061 (5)	0.063 (5)	0.003 (4)	0.004 (4)	0.012 (4)
C34	0.110 (7)	0.073 (6)	0.064 (5)	0.018 (5)	−0.007 (5)	−0.009 (5)
C35	0.059 (6)	0.069 (6)	0.080 (6)	−0.013 (5)	0.009 (5)	−0.009 (5)
C36	0.086 (7)	0.169 (10)	0.078 (6)	0.013 (7)	−0.016 (6)	0.015 (7)
C37	0.076 (6)	0.062 (5)	0.088 (6)	0.005 (5)	0.005 (5)	−0.016 (5)
C38	0.115 (8)	0.192 (10)	0.067 (6)	0.045 (7)	0.038 (6)	0.007 (7)

### Geometric parameters (Å, º)

**Table:**

N1—C7	1.290 (7)	C16—H16B	0.9700
N1—N2	1.392 (7)	C17—H17A	0.9600
N2—C8	1.371 (8)	C17—H17B	0.9600
N2—H2	0.898 (11)	C17—H17C	0.9600
N3—C4	1.355 (8)	C18—C19	1.498 (9)
N3—C16	1.455 (8)	C18—H18A	0.9700
N3—C18	1.497 (8)	C18—H18B	0.9700
N4—C26	1.282 (7)	C19—H19A	0.9600
N4—N5	1.382 (7)	C19—H19B	0.9600
N5—C27	1.342 (8)	C19—H19C	0.9600
N5—H5	0.901 (11)	C20—C25	1.375 (8)
N6—C23	1.378 (7)	C20—C21	1.400 (8)
N6—C35	1.449 (8)	C20—C26	1.444 (8)
N6—C37	1.461 (8)	C21—C22	1.359 (8)
O1—C2	1.369 (7)	C22—C23	1.380 (8)
O1—H1	0.8200	C22—H22	0.9300
O2—C8	1.217 (7)	C23—C24	1.420 (8)
O3—C21	1.365 (7)	C24—C25	1.371 (8)
O3—H3	0.847 (10)	C24—H24	0.9300
O4—C27	1.235 (7)	C25—H25	0.9300
C1—C6	1.392 (8)	C26—H26	0.9300
C1—C2	1.406 (8)	C27—C28	1.492 (8)
C1—C7	1.453 (8)	C28—C33	1.351 (8)
C2—C3	1.379 (8)	C28—C29	1.402 (8)
C3—C4	1.389 (8)	C29—C30	1.385 (8)
C3—H3A	0.9300	C29—H29	0.9300
C4—C5	1.407 (8)	C30—C31	1.379 (8)
C5—C6	1.350 (8)	C30—H30	0.9300
C5—H5A	0.9300	C31—C32	1.389 (9)
C6—H6	0.9300	C31—C34	1.515 (8)
C7—H7	0.9300	C32—C33	1.372 (8)
C8—C9	1.479 (8)	C32—H32	0.9300
C9—C14	1.369 (8)	C33—H33	0.9300
C9—C10	1.378 (8)	C34—H34A	0.9600
C10—C11	1.385 (8)	C34—H34B	0.9600
C10—H10	0.9300	C34—H34C	0.9600
C11—C12	1.368 (9)	C35—C36	1.497 (9)
C11—H11	0.9300	C35—H35A	0.9700
C12—C13	1.394 (9)	C35—H35B	0.9700
C12—C15	1.523 (9)	C36—H36A	0.9600
C13—C14	1.379 (8)	C36—H36B	0.9600
C13—H13	0.9300	C36—H36C	0.9600
C14—H14	0.9300	C37—C38	1.485 (9)
C15—H15A	0.9600	C37—H37A	0.9700
C15—H15B	0.9600	C37—H37B	0.9700
C15—H15C	0.9600	C38—H38A	0.9600
C16—C17	1.424 (7)	C38—H38B	0.9600
C16—H16A	0.9700	C38—H38C	0.9600
			
C7—N1—N2	116.5 (5)	C19—C18—H18B	109.3
C8—N2—N1	117.1 (5)	H18A—C18—H18B	107.9
C8—N2—H2	129 (4)	C18—C19—H19A	109.5
N1—N2—H2	113 (4)	C18—C19—H19B	109.5
C4—N3—C16	119.6 (7)	H19A—C19—H19B	109.5
C4—N3—C18	125.1 (6)	C18—C19—H19C	109.5
C16—N3—C18	114.9 (7)	H19A—C19—H19C	109.5
C26—N4—N5	116.8 (6)	H19B—C19—H19C	109.5
C27—N5—N4	119.6 (6)	C25—C20—C21	115.6 (7)
C27—N5—H5	119 (4)	C25—C20—C26	120.7 (7)
N4—N5—H5	120 (4)	C21—C20—C26	123.7 (7)
C23—N6—C35	122.5 (6)	C22—C21—O3	117.5 (7)
C23—N6—C37	121.6 (6)	C22—C21—C20	122.6 (7)
C35—N6—C37	115.9 (6)	O3—C21—C20	119.7 (7)
C2—O1—H1	109.5	C21—C22—C23	120.7 (7)
C21—O3—H3	120.6 (17)	C21—C22—H22	119.7
C6—C1—C2	116.3 (6)	C23—C22—H22	119.7
C6—C1—C7	121.0 (7)	N6—C23—C22	121.1 (7)
C2—C1—C7	122.7 (6)	N6—C23—C24	120.5 (7)
O1—C2—C3	116.7 (7)	C22—C23—C24	118.4 (7)
O1—C2—C1	121.5 (6)	C25—C24—C23	118.5 (7)
C3—C2—C1	121.7 (7)	C25—C24—H24	120.7
C2—C3—C4	120.1 (7)	C23—C24—H24	120.7
C2—C3—H3A	120.0	C24—C25—C20	124.0 (7)
C4—C3—H3A	120.0	C24—C25—H25	118.0
N3—C4—C3	119.2 (7)	C20—C25—H25	118.0
N3—C4—C5	122.1 (7)	N4—C26—C20	119.7 (7)
C3—C4—C5	118.6 (7)	N4—C26—H26	120.2
C6—C5—C4	120.0 (7)	C20—C26—H26	120.2
C6—C5—H5A	120.0	O4—C27—N5	121.0 (7)
C4—C5—H5A	120.0	O4—C27—C28	122.6 (7)
C5—C6—C1	123.1 (7)	N5—C27—C28	116.3 (7)
C5—C6—H6	118.4	C33—C28—C29	117.6 (6)
C1—C6—H6	118.4	C33—C28—C27	119.8 (7)
N1—C7—C1	121.2 (6)	C29—C28—C27	122.6 (7)
N1—C7—H7	119.4	C30—C29—C28	120.5 (7)
C1—C7—H7	119.4	C30—C29—H29	119.8
O2—C8—N2	122.7 (7)	C28—C29—H29	119.8
O2—C8—C9	123.8 (7)	C31—C30—C29	120.6 (7)
N2—C8—C9	113.5 (6)	C31—C30—H30	119.7
C14—C9—C10	117.6 (7)	C29—C30—H30	119.7
C14—C9—C8	124.7 (7)	C30—C31—C32	118.5 (7)
C10—C9—C8	117.7 (7)	C30—C31—C34	120.3 (8)
C9—C10—C11	121.3 (7)	C32—C31—C34	121.2 (7)
C9—C10—H10	119.3	C33—C32—C31	119.9 (7)
C11—C10—H10	119.3	C33—C32—H32	120.1
C12—C11—C10	121.0 (7)	C31—C32—H32	120.1
C12—C11—H11	119.5	C28—C33—C32	122.9 (7)
C10—C11—H11	119.5	C28—C33—H33	118.6
C11—C12—C13	117.9 (7)	C32—C33—H33	118.6
C11—C12—C15	121.3 (8)	C31—C34—H34A	109.5
C13—C12—C15	120.8 (8)	C31—C34—H34B	109.5
C14—C13—C12	120.3 (7)	H34A—C34—H34B	109.5
C14—C13—H13	119.8	C31—C34—H34C	109.5
C12—C13—H13	119.8	H34A—C34—H34C	109.5
C9—C14—C13	121.7 (7)	H34B—C34—H34C	109.5
C9—C14—H14	119.1	N6—C35—C36	113.7 (6)
C13—C14—H14	119.1	N6—C35—H35A	108.8
C12—C15—H15A	109.5	C36—C35—H35A	108.8
C12—C15—H15B	109.5	N6—C35—H35B	108.8
H15A—C15—H15B	109.5	C36—C35—H35B	108.8
C12—C15—H15C	109.5	H35A—C35—H35B	107.7
H15A—C15—H15C	109.5	C35—C36—H36A	109.5
H15B—C15—H15C	109.5	C35—C36—H36B	109.5
C17—C16—N3	108.2 (8)	H36A—C36—H36B	109.5
C17—C16—H16A	110.1	C35—C36—H36C	109.5
N3—C16—H16A	110.1	H36A—C36—H36C	109.5
C17—C16—H16B	110.1	H36B—C36—H36C	109.5
N3—C16—H16B	110.1	N6—C37—C38	113.9 (7)
H16A—C16—H16B	108.4	N6—C37—H37A	108.8
C16—C17—H17A	109.5	C38—C37—H37A	108.8
C16—C17—H17B	109.5	N6—C37—H37B	108.8
H17A—C17—H17B	109.5	C38—C37—H37B	108.8
C16—C17—H17C	109.5	H37A—C37—H37B	107.7
H17A—C17—H17C	109.5	C37—C38—H38A	109.5
H17B—C17—H17C	109.5	C37—C38—H38B	109.5
N3—C18—C19	111.8 (7)	H38A—C38—H38B	109.5
N3—C18—H18A	109.3	C37—C38—H38C	109.5
C19—C18—H18A	109.3	H38A—C38—H38C	109.5
N3—C18—H18B	109.3	H38B—C38—H38C	109.5

### Hydrogen-bond geometry (Å, º)

**Table:**

*D*—H···*A*	*D*—H	H···*A*	*D*···*A*	*D*—H···*A*
N2—H2···O4^i^	0.90 (1)	1.95 (2)	2.831 (7)	167 (6)
O1—H1···N1	0.82	1.93	2.641 (7)	145
N5—H5···O2	0.90 (1)	2.12 (2)	2.985 (7)	160 (6)
O3—H3···N4	0.85 (1)	1.94 (1)	2.581 (7)	132 (2)

Symmetry code: (i) *x*+1, *y*, *z*.
